# Supplementary material for: Predicting Antimicrobial Peptide Activity: A Machine Learning-Based Quantitative Structure–Activity Relationship Approach
Source: Pharmaceutics. 2025 Jul 31;17(8):993. doi: 10.3390/pharmaceutics17080993 (PMC12389201; doi:10.3390/pharmaceutics17080993)
Supplement: Supplementary file 1 [file pharmaceutics-17-00993-s001.zip › Figures S1-S15.pdf]

Predicting Antimicrobial Peptide Activity: A Machine Learning-Based Quantitative Structure–Activity Relationship Approach

Eliezer I. Bonifacio-Velez de Villa, María E. Montoya-Alfaro, Luisa P. Negrón-Ballarte and Christian Solis-Calero *

Faculty of Pharmacy and Biochemistry, Universidad Nacional Mayor de San Marcos, Lima 15001, Peru ; eliezerbvv1@gmail.com (E.I.B.-V); mmontoyaa@unmsm.edu.pe (M.E.M.-A.); lnegronb@unmsm.edu.pe (L.P.N.-B.)

*Correspondence: csolisc@unmsm.edu;

Supplementary Materials

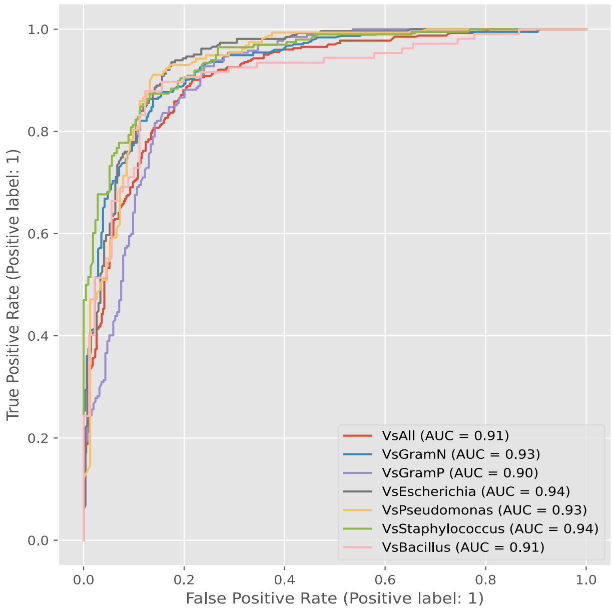

Figure S1: ROC curve of the Random Forest -based classification models on the test dataset during validation.

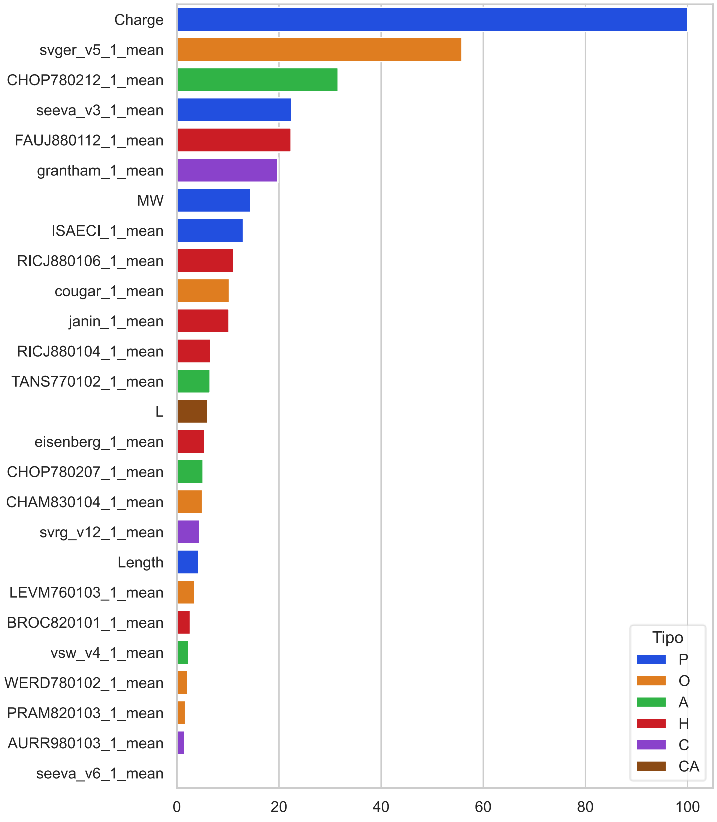

Figure S2: Importance of descriptors in the classification model against all microorganisms

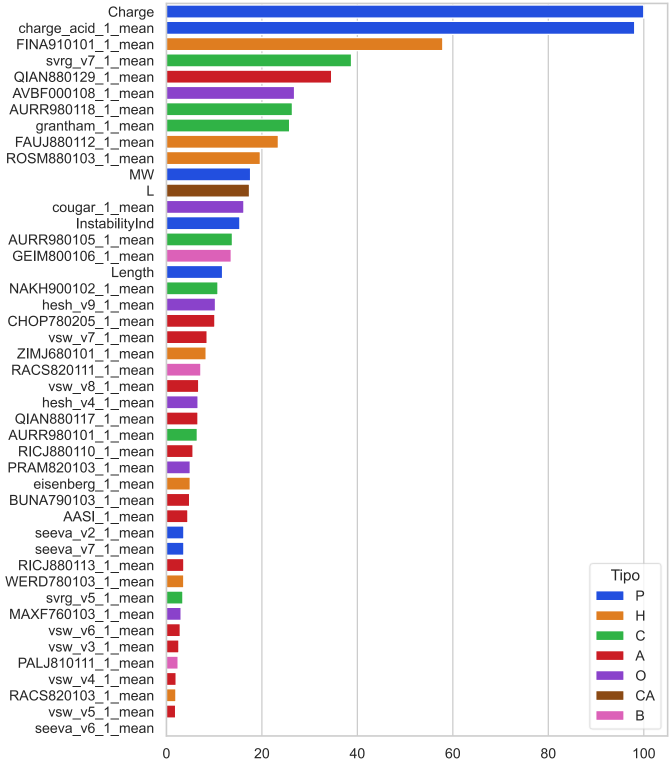

Figure S3: Importance of descriptors in the classification model against Gram-negative organisms

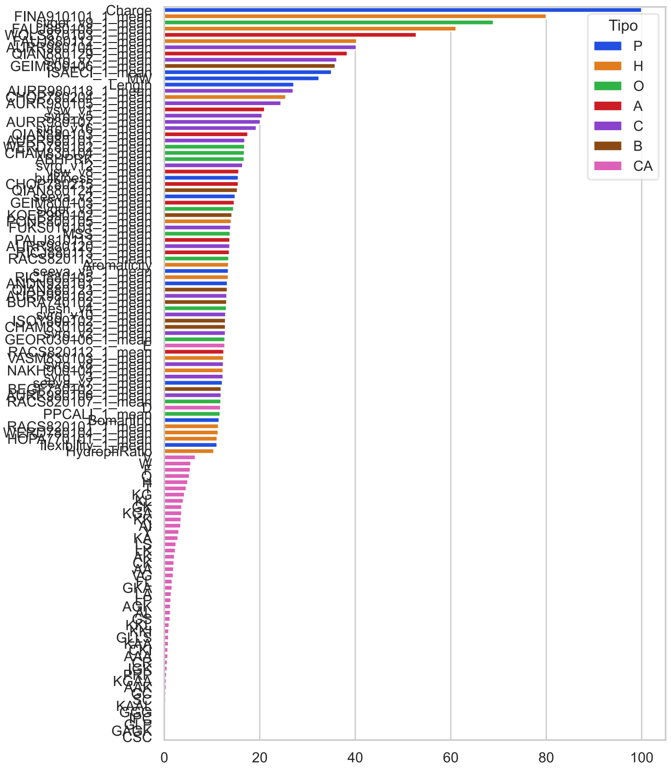

Figure S4: Importance of descriptors in the classification model against Gram-positive organisms

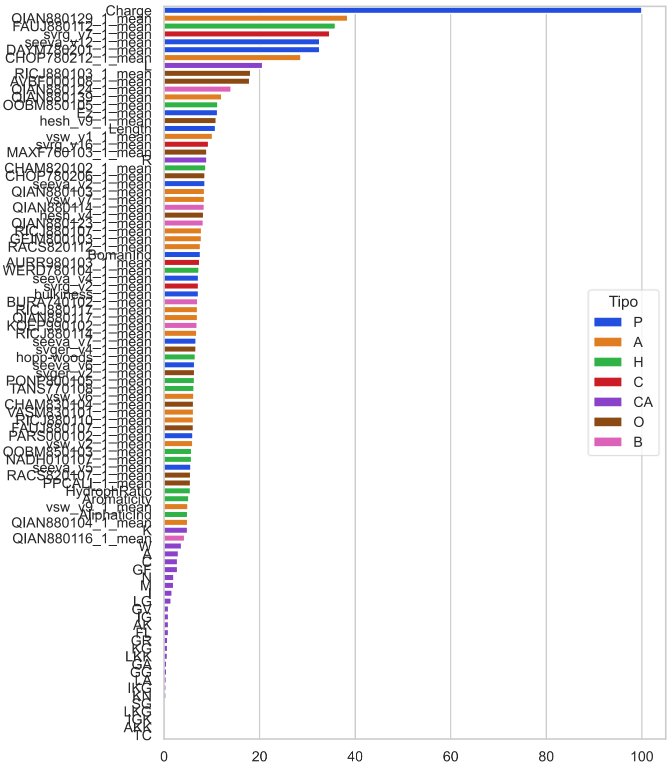

Figure S5: Importance of descriptors in the classification model against organisms of the genus *Escherichia*

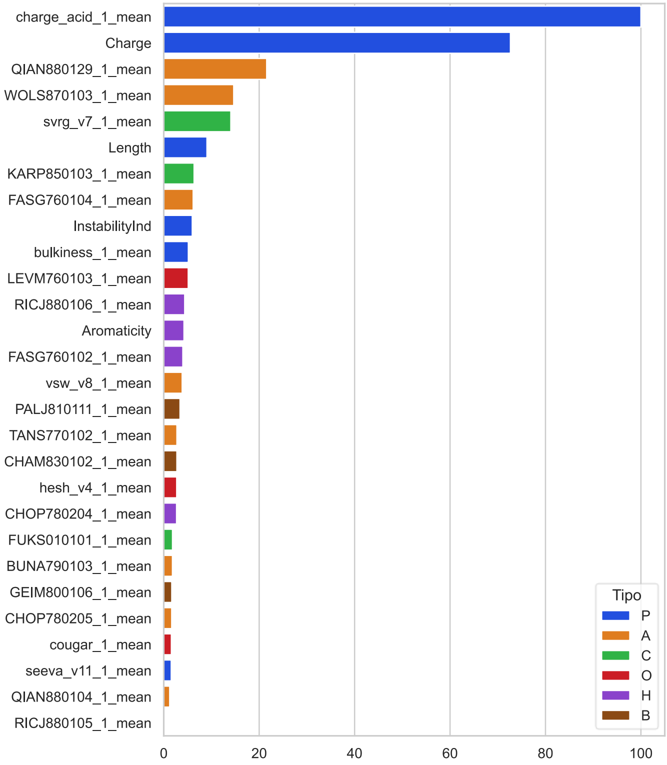

Figure S6: Importance of descriptors in the classification model against organisms of the genus *Pseudomonas*

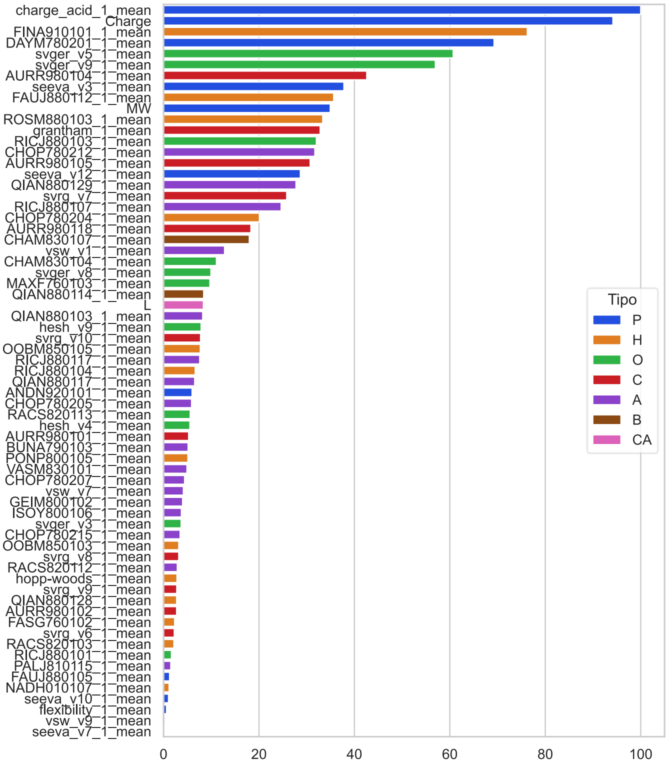

Figure S7: Importance of descriptors in the classification model against organisms of the genus *Staphylococcus*

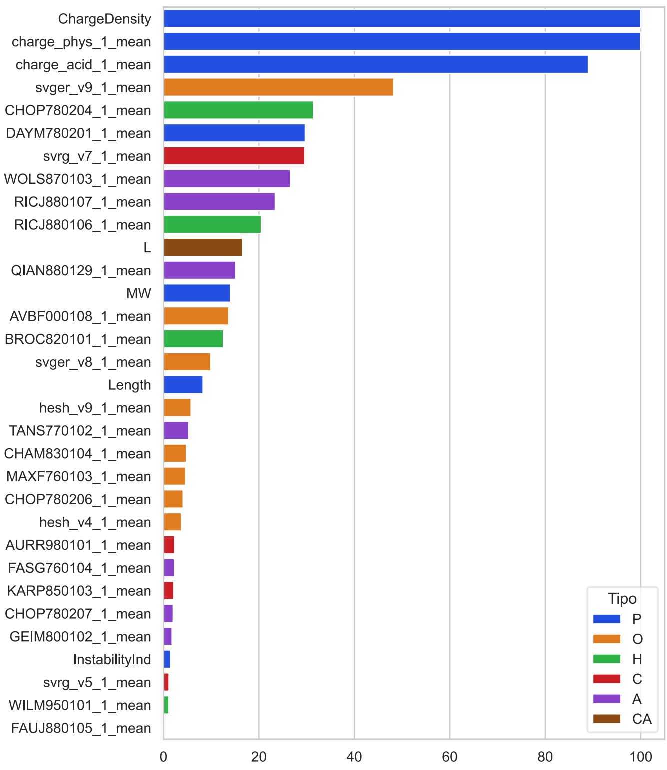

Figure S8: Importance of descriptors in the classification model against organisms of the genus *Bacillus*

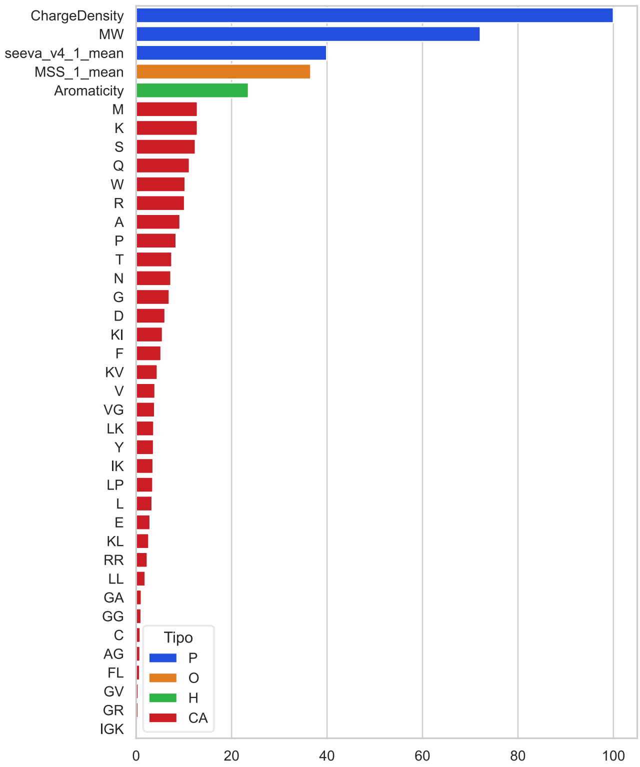

Figure S9: Importance of descriptors in the regression model against all microorganisms

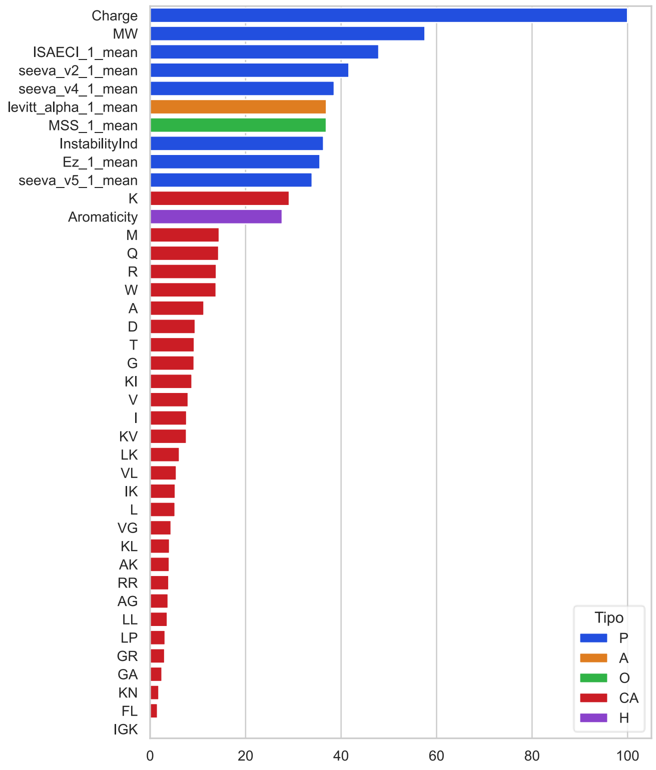

Figure S10: Importance of descriptors in the regression model against Gram-negative organisms

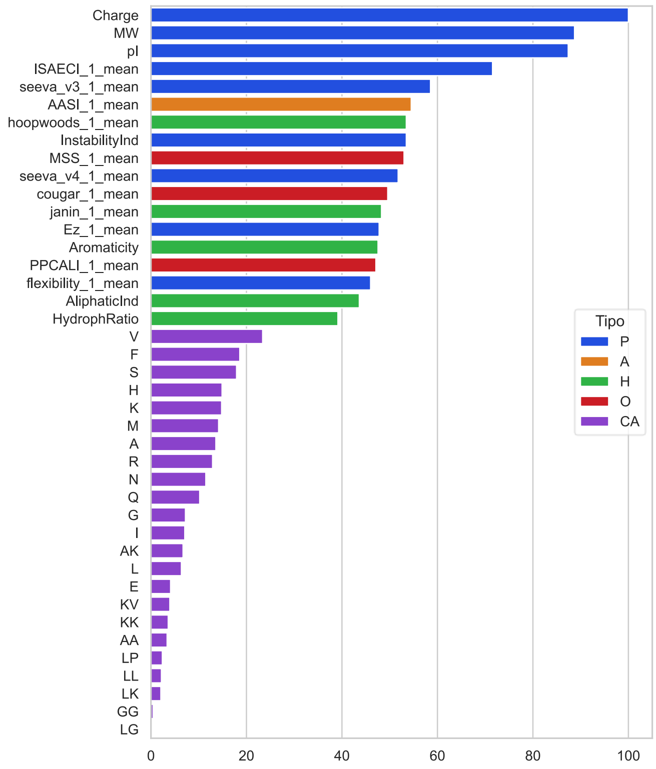

Figure S11: Importance of descriptors in the regression model against Gram-positive organisms

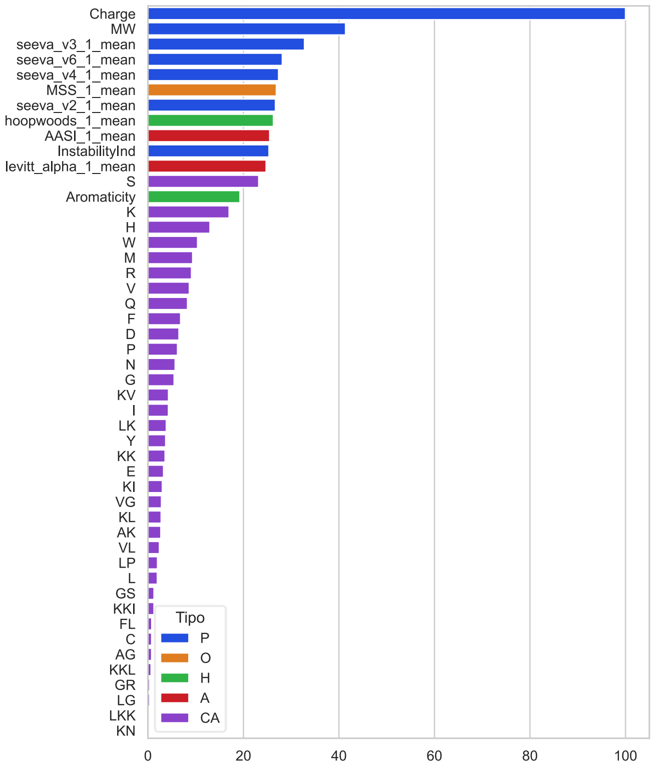

Figure S12: Importance of descriptors in the regression model against organisms of the genus *Escherichia*

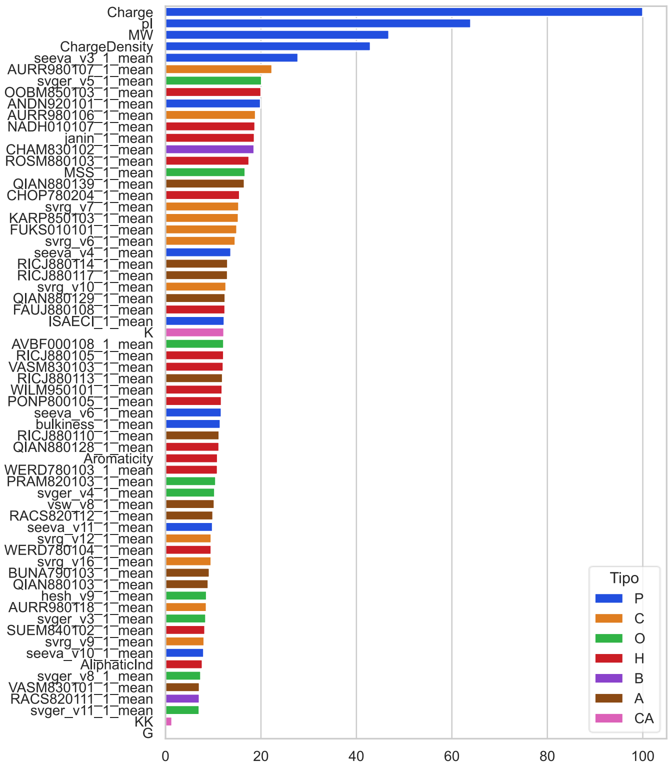

Figure S13: Importance of the descriptors in the regression model against organisms of the genus *Pseudomonas*

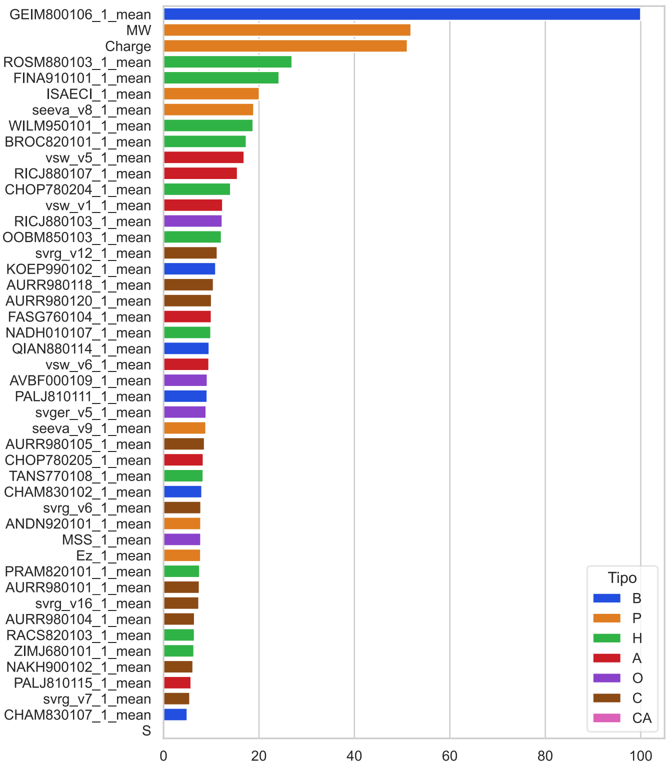

Figure S14: Importance of the descriptors in the regression model against organisms of the genus *Staphylococcus*

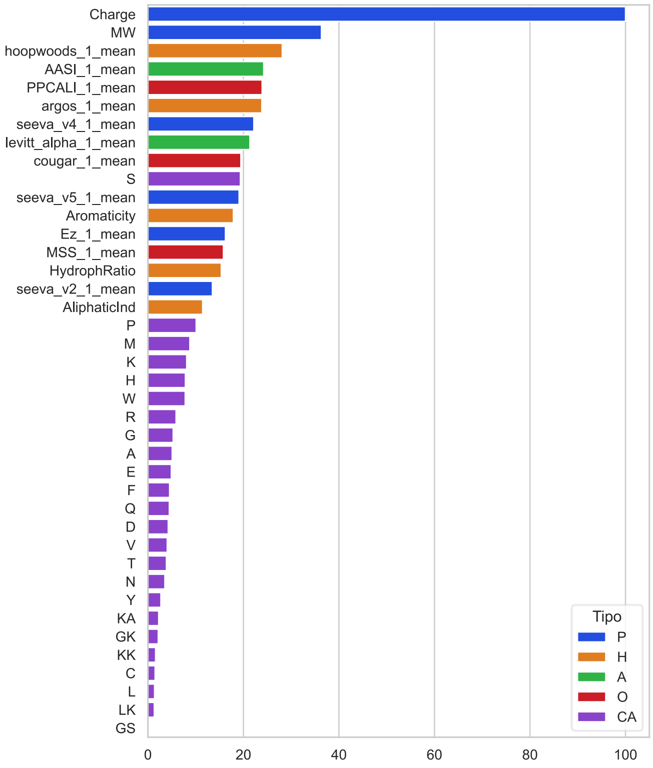

Figure S15: Importance of the descriptors in the regression model against organisms of the genus *Bacillus*.
